# A Text-Book on Nervous Diseases

**Published:** 1895-12

**Authors:** 


### Book Reviews.

A TEXT-BOOK ON NERVOUS DISEASES. By American authors. Edited by F. X. DERCUM, M. D., Clinical Professor of Diseases of the Nervous System in the Jefferson Medical College, Philadelphia. In one handsome octavo volume of 1052 pages, with 341 engravings and seven colored plates. Cloth, $6.00 ; leather, $7.00. Philadelphia : Lea Brothers & Co., Publishers. 1895.

This work has for contributors some of the most prominent men in New York, Philadelphia and other centers. The arrangement of the subjects is good and somewhat different from that usually followed in similar works.

The opening chapter, by Weir Mitchell and the editor, on general considerations, contains some new diagrams and observations. Among the illustrations of newer instruments of precision in diagnosis we notice with pleasure Krauss's foot and leg dynamometer.

The examination of the eye from a neurological standpoint is is by Charles A. Oliver. The general morbid states of the nervous system are taken up by the editor, J. H. Lloyd and P. C. Knapp, in three chapters.

The chapter on traumatic neurosis is particularly full and complete. Under this heading we are interested in the prognosis given by the author—namely, "It is probable that a large proportion of the cases, taking all cases as they come, if they could be placed under proper conditions immediately after the injury, would do well." E. D. Fisher discusses the diathetic and toxic affections of the nervous system, in a series of short, pithy articles. Chapter VI. deals with diseases the direct or indirect result of infection, by Osler. Wharton Sinkler treats of choreiform affections, in a chapter in which more than the usual space is allotted to this subject. It is rendered very plain by the numerous illustrations, mostly photographic, with which it is embellished. Local and occupation spasms is by C. W. Burr, in which we notice some varieties which have not previously received much attention in neurological works. Functional tremors, paralysis agitans and epilepsy are by Gray. In connection with epilepsy he gives a very complete table of the results, immediate and permanent, of cranial operation cases. We note that out of forty-four cases operated upon only two are given as cured. General diseases of the brain are written about by N. E. Brill and the editor.

The anatomy of the cerebral cortex and the localisation of its functions, by Charles K. Mills, is one of the most interesting chapters and largely illustrated. We notice that Mills, in his article on sensory localisation, still maintains, as in 1888, that "There is in the cerebrum a region for general sensation, including touch, pain, temperature and, possibly, the senses of pressure and the location of a limb, which could be divided into special sub-areas for the various distinct portions of the body, and that these regions lay alongside of and in close relation with corresponding motor areas, but that they were not identical with them," also that no part of the brain was more likely to contain these differentiated areas for sensation than the gyrus fornicatus, the hippocampal gyre, the precuneus and the post parietal convolutions. In reference to visual localisation there is not yet harmony in the entire cerebral representation of the retina. Of auditory localisation he says: "The localisation of the auditory sphere can be regarded as settled as being in the superior temporal gyres." The olfactory center can best be located in the uncirate gyres. Gustatory localisation the author locates in the fourth temporal convolution. Of diseases in the prefrontal region he says:

> Mental disturbances of a peculiar character occur, such as mental slowness and uncertainty, want of attention and control, and impairment of judgment and reason; closely studied the inhibitory influence of the brain both upon the psychical and physical action, appears to be diminished. Memory is not seriously affected, although a continuous train of thought cannot well be followed and complex intellectual processes cannot be thoroughly performed.

Focal diseases of the brain, by Charles L. Dana, Allen Starr and the editor, occupy three chapters that are profusely illustrated. The diseases of the spinal cord are completely and clearly considered by Lloyd, Prince and Peterson. Paretic dementia is by the editor, also syphilis of the nervous system, the latter being a welcome addition to that branch of medical literature; the diseases of nerves in general, by Wharton Senkler; diseases of the cranial nerves, by George E. de Schweinitz and Herter; diseases of the muscles, by Jacobi; tropho-neuroses, by Collins, are all complete and profusely illustrated. We would especially call attention to the collection of photographs of cretins shown.

We notice with interest the positive statement in treatment of exophthalmic goitre, "The sheep's thyroid gland and its extract are useless." The article on headache is scanty and incomplete. We doubt if a person unacquainted with the subject would find very much help here.

The surgery of the brain, spinal cord and nerves is a complete *résumé* of this subject up to date. We could only wish it had been a little more extended. The book closes with a few general considerations on neuro-electro therapeutics, by George W. Jacoby. The volume will certainly meet the demand for a treatise on nervous diseases up to date, but we express the hope that for some time to come books on this subject will partake more of the monographic type. J. W. P.

